# Neurons in the monkey orbitofrontal cortex mediate reward value computation and decision-making

**DOI:** 10.1038/s42003-019-0363-0

**Published:** 2019-04-05

**Authors:** Tsuyoshi Setogawa, Takashi Mizuhiki, Narihisa Matsumoto, Fumika Akizawa, Ryosuke Kuboki, Barry J. Richmond, Munetaka Shidara

**Affiliations:** 1 Faculty of Medicine, University of Tsukuba, 1-1-1 Tennodai, Tsukuba, Ibaraki 305-8577 Japan; 2 Department of Health and Human Services, Laboratory of Neuropsychology, National Institute of Mental Health, National Institutes of Health, Bethesda, MD 20892-4415 USA; 3 Doctoral Program in Kansei, Behavioral and Brain Science, Graduate School of Comprehensive Human Sciences, University of Tsukuba, 1-1-1 Tennodai, Tsukuba, Ibaraki 305-8577 Japan; 4 Human Informatics Research Institute, AIST, 1-1-1 Umezono, Tsukuba, Ibaraki 305-8568 Japan

## Abstract

Choice reflects the values of available alternatives; more valuable options are chosen more often than less valuable ones. Here we studied whether neuronal responses in orbitofrontal cortex (OFC) reflect the value difference between options, and whether there is a causal link between OFC neuronal activity and choice. Using a decision-making task where two visual stimuli were presented sequentially, each signifying a value, we showed that when the second stimulus appears many neurons encode the value difference between alternatives. Later when the choice occurs, that difference signal disappears and a signal indicating the chosen value emerges. Pharmacological inactivation of OFC neurons coding for choice-related values increases the monkey’s latency to make a choice and the likelihood that it will choose the less valuable alternative, when the value difference is small. Thus, OFC neurons code for value information that could be used to directly influence choice.

## Abstract (web summary)

Tsuyoshi Setogawa et al. identify a population of neurons within the monkey orbitofrontal cortex that encode the difference in value between two offered options. These results suggest a causal role for the monkey orbitofrontal cortex in coding value information to influence decision-making.

## Introduction

When faced with having to choose between two alternatives, the one chosen most often is deemed most valuable. By comparing pairs of alternatives, the alternatives can be ranked in value. This relative value reflects the combination of parameters often measured on different scales, such as reward size and delay or physical work to reward delivery, so a smaller reward that is delivered immediately might be equal in value to a larger reward delivered after a delay or after having to work. In value-based decision-making, three steps are needed to choose an alternative: (1) value encoding of each alternative, (2) comparing these values, and (3) making the choice. The question arises about where in the brain the first two steps occur.

One strong candidate for the site where value is calculated and connected to the choice is the orbitofrontal cortex (OFC). Orbitofrontal neurons are known to carry signals about the expected reward amount and reward type from presented options^1–9^. They are also modulated in relation to the amount of time or physical work needed to obtain the reward^8–10^. Previous behavioral and imaging studies in humans and non-human primates have suggested that the OFC is involved in comparing reward values^11,12^.

In previous neurophysiological studies about value-based decision-making, choice options have been presented simultaneusly^2,3,6,8,9^. To disentangle neuronal activity related to the value comparison between offered alternatives, we developed a decision-making task in which the visual stimuli indicating both the reward size and the work needed to get the reward^13^ were presented sequentially before choice. Separating the visual stimuli in time made it straightforward to analyze the relation between the value of the first stimulus presented in a trial and the value’s relation to the neuron’s firing. When the second stimulus is presented, this task allows us to examine whether the OFC neurons encode a difference in value of offered alternatives, which could be used for value comparison.

Here we recorded single neurons in area 13 of the OFC while rhesus monkeys (*Macaca mulatta*) carried out our decision-making task. We asked: (1) whether and how the reward values are encoded in the OFC neuron, and (2) whether the OFC inactivation affects the choice behavior. Many OFC neurons encoded the reward value of the alternatives calculated from different parameters, that is, reward size and workload. By examining the neuronal activity in the second target presentation period, we found many neurons relating to the value difference as well as neurons encoding the reward value of the currently presented choice target. Reversible OFC inactivation with muscimol at a locus, where we recorded neurons encoding information about reward value caused degradation of the choice performances when the two choice options were close in value. These findings suggest that OFC neurons play a causal role in driving choices, and that this tissue is critical for making fine distinctions in reward value.

## Results

**Fig. 1:**
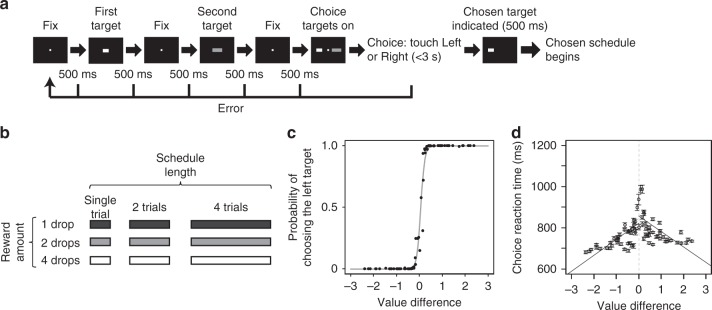
Task procedure and model fitting of choice. **a** Sequence of the decision-making part of the task. By touching the center bar, two different choice targets were sequentially presented. Then, these two targets reappeared simultaneously one on each side of a fixation spot in random order (choice phase). The monkey could choose one of the alternatives by touching a bar on the side corresponding to the choice. After choosing one of them, the chosen reward schedule began (see Methods section). **b** Choice target set. The brightness and the length of choice targets were proportional to the reward amount and schedule length, respectively. **c** Model fitting of choice behavior. To estimate the reward value of each choice target, the monkeys’ choice probability was fit by the exponential discounting model (Eq. 8). Black dots: actual choice data of monkey P (all recorded trials: *n* = 40,625), gray line: the estimated choice probability by model fitting (Generalized linear model (Eq. 12), *β*_1_: *z* = 69.88, *p* < 2.0 × 10^−16^). **d** Choice reaction time vs value difference of left and right choice targets. Circles indicate the mean of reaction times for each value difference and the error bars are SEM. The left and right lines were drawn by linear regression using reaction time data when right and left target was chosen, respectively

We trained two monkeys to perform a decision-making schedule task. Each trial had a decision-making part and a subsequent reward schedule part. In the decision-making part, two choice targets were presented sequentially (Fig. 1a). The target brightness indicated the reward amount (1, 2, or 4 drops of liquid reward) and the target length indicated the workload (1, 2, or 4 visual discrimination trials) (Fig. 1b). These targets then reappeared simultaneously one on each side of a fixation spot. At this time, the monkey chose one of the alternatives by touching a bar on the side corresponding to the choice. Then the monkey had to complete the chosen reward schedule, i.e., the number of trials to work to obtain the indicated amount of reward^14–18^ (Supplementary Fig. 1). This design allowed us to analyze (1) how the target values were represented by the neuronal activity during the first target presentation period, (2) whether and how the values of the two targets were coded by the neuronal activity during the second target presentation period. We recorded 256 OFC neurons from two monkeys (monkey P: 137, monkey H: 119) and investigated the relationship between neuronal activities and estimated reward value of alternatives.

### Behavioral results and reward value estimation

In most trials, the monkeys chose what seemed intuitively to us to be higher value schedules, i.e., targets indicating shorter schedules with larger rewards (Supplementary Fig. 2), showing that the monkeys were cognizant of the choice targets.

In this design there is an interplay between schedule length and reward size, e.g., how is a large reward with a larger workload valued? A standard way to estimate the subjective value of alternatives when there are intermediate combinations of incentives (and disincentives) such as reward size and schedule length is to fit a discounting function to the performance data^19–21^. Here, we fit the choice data using exponential discounting of reward value with one free fitting parameter, the discount factor *k* (Eq. 8 in Methods section). We estimated the discounting factor, *k*, for every recording session using these reward values and the monkeys’ choices (see Methods) (mean ± SD *k* value; monkey P: 0.46 ± 0.05 (137 sessions), monkey H: 0.48 ± 0.11 (119 sessions)) (Fig. 1c for monkey P). We also examined the relation between choice reaction time and value difference for the left and right choice targets separately (the monkeys were faster when responding to the target on the side of the hand used). The reaction time increased as the difference in value between two choice targets became smaller (Fig. 1d for monkey P) (linear regression, the trials when the animal chose the left side target in the choice phase; monkey P: *n* = 19,424, *R*^2^ = 0.06, *t* = −297, *p* < 2.0 × 10^−16^, monkey H: *n* = 18,322, *R*^2^ = 0.02, *t* = −146, *p* < 2.0 × 10^−16^; the trials when the animal chose the right side target in the choice phase; monkey P: *n* = 21,201, *R*^2^ = 0.06, *t* = 307, *p* < 2.0 × 10^−16^, monkey H: *n* = 18,917, *R*^2^ = 0.03, *t* = 166, *p* < 2.0 × 10^−16^). Thus, it appears that the animals are sensitive to the values of presented targets for all pairings of the choice alternatives. The choice probability increased monotonically with increasing subjective value (Supplementary Fig. 3) and was unbiased with respect to whether the most valuable target was presented first or second (Generalized linear model (GLM), monkey P: df = 17, *z* = 0.13, *p* = 0.90; monkey H: df = 17, *z* = −0.19, *p* = 0.85) (Eq. 13). When the largest or smallest value target (4–1 [drop/schedule length] or 1–4) was presented as the first target, the animal could have reached a decision before the second target was presented. Therefore we analyzed the relation between the choice reaction time and the value of the second target when the largest or smallest value target was presented as the first target. If the monkey reached a decision using only the first target value, the choice reaction time would be flat regardless of the second target value. However, the relation between the choice reaction time and the value of the second target showed significant linear relation (linear regression, [4–1] monkey P: *p* < 2.0 × 10^−16^, monkey H: *p* < 2.0 × 10^−16^, [1–4] monkey P: *p* < 2.0 × 10^−16^, monkey H: *p* < 2.0 × 10^−16^) (Supplementary Fig. 4). This result suggests that the animals paid attention to the second target even when the first target was the largest or the smallest value target.

### OFC combines different dimensions into a single reward value

**Fig. 2:**
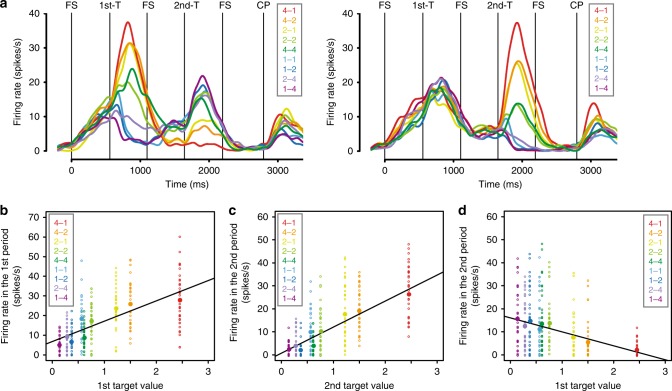
An example of value-related neuronal response (value difference neuron). **a** Left and right panels show the responses classified by the first and the second target, respectively. The reward amount and the schedule length of the choice target are abbreviated as “drop – trial”, and the colors correspond to the response in each target. These labels are ordered according to the estimated reward value. FS, fixation spot; 1st-T, first target; 2nd-T, second target; CP, choice phase. **b** Relationship between the first target value and the firing rate during the first target presentation period (GLM (Eq. 17), *n* = 340, *R*^2^ = 0.30, *θ*_1_: *t* = 13.89, *p* < 2.0 × 10^−16^). Dots: trials, filled large circles: average activity in each target value. **c**, **d** Relationship between the firing rate during the second target presentation period and **c** the second target value (GLM, *n* = 340, *R*^2^ = 0.48, *α*_1_: *t* = 17.63, *p* < 2.0 × 10^−16^) or **d** the first target value (GLM, *n* = 340, *R*^2^ = 0.13, *α*_1_: *t* = −7.29, *p* < 2.2 × 10^−12^). The same conventions as in **b**

The neuronal activity was compared to the target values calculated from the discounting model for the behavior during the recording sessions. Figure 2 shows an example of value-related neuron’s responses in both the first and the second target periods. The activity of this neuron was related to the value of the presented target (Fig. 2a, b). About 70% of the neurons showed a relation to the value of the presented target in the first target presentation period (173/256, 67.6%, GLM, *p* < 0.05) (Eq. 17). About 40% (108/256) of the neurons showed responses that were correlated with both the reward amount and the workload (42.2%, GLM, *p* < 0.05) (Eq. 18). About 20% (52/256) of the neurons had responses that were correlated with the reward amount only (20.3%, GLM, *p* < 0.05) and about 15% (43/256) of the neurons had responses that were correlated with the workload only (16.8%, GLM, *p* < 0.05) (Eq. 18). Among 173 neurons showing correlation with target value, 100 (57.8%) showed better fitting by target value rather than reward amount and/or workload (see Methods section). Thus, it appears that this latter group of neurons are sensitive to the discounted value even when it is computed from different dimensions affecting reward, here reward size and workload.

### Target value encoding in OFC neurons

Our interest is in learning how information that supports choice is encoded. In the second target presentation period, the subject had seen both the first and the second target, and therefore presumably had assigned them values. Thus, the subject had the information needed to make a choice in this period. To evaluate the relations between the neuronal activity and the target values, we carried out a model selection procedure using the results from seven analytic models:

1 $${\mathrm{SC}}_{2{\mathrm{nd}}} = \alpha_{0} + \alpha_{1}V_{1},$$

2 $${\mathrm{SC}}_{2{\mathrm{nd}}} = \alpha_{0} + \alpha_{1}V_{2},$$

3 $${\mathrm{SC}}_{2{\mathrm{nd}}} = \alpha_{0} + \alpha_{1}\left( V_{1} + V_{2} \right),$$

4 $${\mathrm{SC}}_{2{\mathrm{nd}}} = \alpha_{0} + \alpha_{1}\left( V_{1} - V_{2} \right),$$

5 $${\mathrm{SC}}_{2{\mathrm{nd}}} = \alpha_{0} + \alpha_{1} {\mathrm{CT}},$$

6 $${\mathrm{SC}}_{2{\mathrm{nd}}} = \alpha_{0} + \alpha_{1} {\mathrm{CT}}_{\mathrm{V}},$$

7 $${\mathrm{SC}}_{2{\mathrm{nd}}} = \alpha_{0} + \alpha_{1}\left( {{\mathrm{CT}}_{\mathrm{V}} - {\mathrm{unCT}}_{\mathrm{V}}} \right),$$

where SC_2nd_ is the spike count during the second target presentation period and the dependent variable, *α*_0_ is the intercept, *α*_1_ is the coefficient estimated by linear regression, *V*_1_ and *V*_2_ are the first and the second target value, CT is the chosen target (the first target or the second target), and CT_V_ and unCT_V_ are the chosen target value and unchosen target value, respectively. We tested these models by using GLM with a Poisson link function.

Models 1 and 2 relate the neuronal response at the time of the second stimulus appearance to the first or the second target value alone. Models 3 and 4 relate the neuronal response to the value summation and value difference between two choice targets, respectively. Model 5 relates the neuronal response to the chosen target (the first target or the second target). Model 6 relates the neuronal activity to the chosen target value, and model 7 relates the activity to the difference between the chosen and unchosen target values. Neurons have been analyzed using models equivalent to 1, 2, 5, 6, and 7 previously^3,5,10^. We classified the neuronal selectivity using akaike information criterion (AIC), with the model having the smallest AIC taken to describe the neuronal selectivity.

**Fig. 3:**
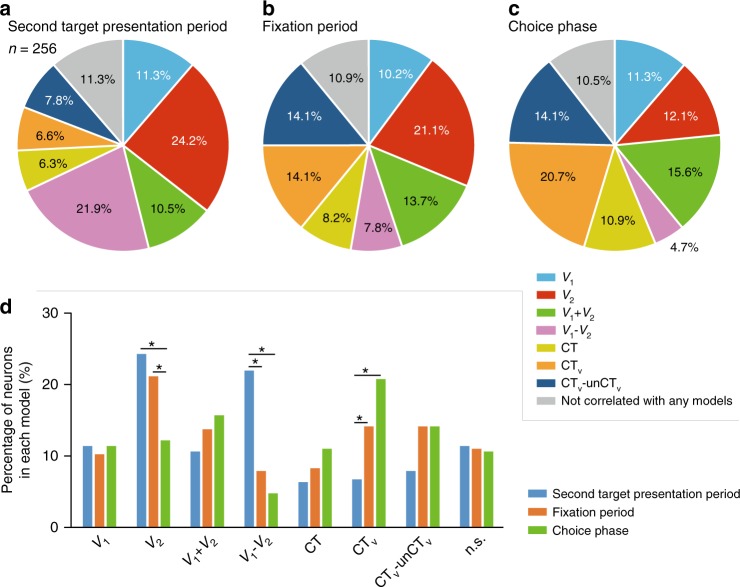
Model classification of recorded neurons. **a** Model classification in the second target presentation period. **b** Model classification in the fixation period after the second target presentation period. **c** Model classification in the choice phase. **d** Percentage of neurons in each model. Eqs. 1–7 were used for these model selections. *V*_1,_ first target value; *V*_2_, second target value; CT, chosen target; CT_V,_ chosen target value; unCT_V_, unchosen target value. *p < 0.01 (*χ*^2^-test, Bonferroni correction)

Figure 2 illustrates a neuron where model 4 fit best. Such a neuron reflects a value comparison between offered alternatives because the firing rate in the second target presentation period shows the negative (positive) correlation with the first target values and positive (negative) correlation with the second target values (Fig. 2c, d). Figure 3a shows that the top two largest groups of responsive neurons were fit best by the model relating to value difference between offered alternatives and value of the currently presented target (model 4: 56/256, 21.9%; model 2: 62/256, 24.2%, Fig. 2 and Supplementary Fig. 5 for an example neuron, respectively). There were not many neurons with selectivity for the other models (model 1: 29/256, 11.3%; model 3: 27/256, 10.5%; model 5: 16/256, 6.3%; model 6: 17/256, 6.6%; model 7: 20/256, 7.8%). These results suggest that the main roles of the OFC neurons in the second target presentation period are estimating the value of the presented choice target and calculating the difference in estimated value between the two choice targets.

To examine the transition from selectivity in the neuronal responses during the second target presentation period (Fig. 3a) to the selectivity in the choice phase (Fig. 3c), we also analyzed the neuronal activity in the fixation period, i.e., the period just after the second target disappeared, and in the choice period. At the beginning of the fixation period the selectivity in the population shifted. The number of neurons with selectivity for the chosen value increased from 17/256 (6.6%) to 36/256 (14.1%), becoming greatest in the choice phase 53/256 (20.7%) (Fig. 3). The number of neurons relating to the value difference became small immediately after the second target was extinguished (fixation period: 20/256, 7.8%; choice phase: 12/256, 4.7%) (Fig. 3b–d). Many neurons that were selective for the chosen value in the choice phase were different from the neurons that were selective for the value difference in the second target presentation period (Table 1).

**Table 1:** Number of neurons in each model in the second target presentation period and the choice phase

		Choice phase								Total
		*V*_1_	*V*_2_	*V*_1_ + *V*_2_	*V*_1_ − *V*_2_	CT	CT_v_	CTv-unCT_v_	n.s.	
**2nd target presentation period**	***V***_**1**_	3	4	3	5	2	5	5	2	29
	***V***_**2**_	5	8	15	1	6	15	6	6	62
	***V***_**1**_ + ***V***_**2**_	3	2	4	0	3	9	2	4	27
	***V***_**1**_ − ***V***_**2**_	12	7	7	5	7	9	6	3	56
	**CT**	2	5	1	1	3	1	2	1	16
	**CT**_**v**_	2	0	1	0	0	9	4	1	17
	**CT**_**v**_-**unCT**_**v**_	0	1	2	0	3	2	8	4	20
	**n.s.**	2	4	7	0	4	3	3	6	29
**Total**		29	31	40	12	28	53	36	27	256

### OFC inactivation causes degradation of choice behavior

**Fig. 4:**
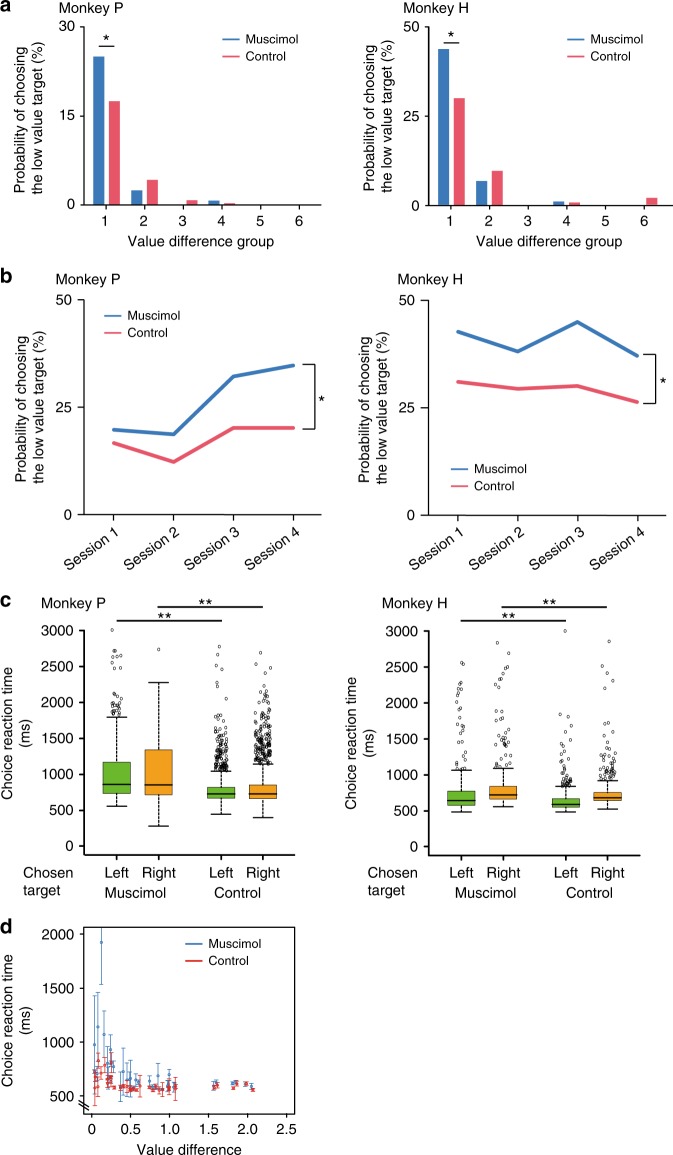
Effects of pharmacological OFC inactivation on choice performance. **a** Relationship between the choice probability of choosing the low value target and the difference in value. The difference in values were ordered from lowest to highest, and then these values were divided into six groups according to the value difference (Each group has data of 6 combinations of alternatives. Group 1 was the smallest value difference group. See Supplementary Table 1 and 2). *p < 0.05 (Proportion test, FDR correction). **b** Session-by-session analysis of the probability of choosing the low value target in the smallest value difference group (Group 1 in **a**). *p < 0.05 (GLMM). **c** Choice reaction time. Green and orange boxplots indicate the reaction time for the choice of the left and the right side target, respectively. Circles; outliers, **p < 0.01. **d** Relationship between the value difference and the choice reaction time (choice of the left target in monkey H). Error bar: SEM

If the value coding by these OFC neurons is critical for making good choices, choice behavior should be degraded by inactivating these neurons. We injected muscimol (5 μg/μl, dissolved in normal saline) locally at almost symmetrical locations in area 13 of both hemispheres (Supplementary Fig. 6). The location was chosen to be where the value-related neurons were recorded while the monkey performed this task. The behavioral data were compared to sessions with only saline injection. Consistent with the previous OFC lesion study^22^, the choice was affected with muscimol treatment (Generalized linear mixed model (GLMM, Eq. 14); [*γ*_1_] *z* = −11.3, *p* < 2.0 × 10^−16^; [*γ*_2_] *z* = −2.62, *p* = 8.9 × 10^−3^; [interaction] *z* = 2.09, *p* = 0.04). Because there was a significant interaction between the value difference and muscimol treatment, we analyzed the relationship between the difference in value and the probability of choosing the low value target. For both monkeys, the ratio of low value targets chosen increased with muscimol treatment when the difference in value between two alternatives was small (Proportion test, monkey P: *Z* = 2.33, *p* = 2.0 × 10^−2^; monkey H: *Z* = 2.91, *p* = 1.8 × 10^−3^, FDR correction) (Group 1 of Fig. 4a, Supplementary Table 1 and 2). We then analyzed the monkeys’ choice in the smallest value difference group (Group 1 in Fig. 4a) at session-by-session. For both monkeys, the proportion of low value targets chosen in the muscimol condition was significantly larger than in the control even at the session level (GLMM, Eq. 15; monkey P: [*ρ*_1_] *z* = −3.45, *p* = 5.5 × 10^−4^; [*ρ*_2_] *z* = −2.29, *p* = 2.2 × 10^−2^; monkey H: [*ρ*_1_] *z* = −6.46, *p* = 1.0 × 10^−10^; [*ρ*_2_] *z* = −2.77, *p* = 5.5 × 10^−3^) (Fig. 4b).

We also analyzed the choice reaction time in the choice phase. In both monkeys the choice reaction times became longer in the muscimol condition (two-tailed *t*-test, monkey P: [left] *t* = −13.26, df = 1081.41, *p* < 2.2 × 10^−16^, [right] *t* = −11.71, df = 1335.58, *p* < 2.2 × 10^−16^; monkey H: [left] *t* = 4.73, df = 448.97, *p* = 3.0 × 10^−6^, [right] *t* = 3.73, df = 534.22, *p* = 2.1 × 10^−4^) (Fig. 4c, d, Supplementary Table 3). There was an interaction between the value difference and treatments (GLMM, Eq. 16; [*τ*_1_] *t* = −9.83, *p* < 2.0 × 10^−16^; [*τ*_2_] *t* = −6.42, *p* = 1.5 × 10^−10^; [*τ*_3_] *t* = 0.08, *p* = 0.94; [interaction] value difference: treatment, *t* = 4.58, *p* = 4.7 × 10^−6^) showing that the reaction times in the muscimol condition depended on the size of the value difference differently than in the saline condition. This can be seen by examining Fig. 4d where at small value differences in the muscimol condition the reaction times were very much larger than those in the saline condition whereas at larger value differences the reaction times in the muscimol condition were about the same as those in the saline condition. If the muscimol inactivation were affecting motor performance directly, we would expect the same effect across all values. However, the inactivation seems to interfere with the willingness to choose mainly, if not exclusively, when the difference in offered target values is small. Thus it seems that the change in choice performance during muscimol treatment is caused by a decrease in the monkeys’ sensitivities to differences in value, that is, when the value difference is small the monkeys have trouble judging which is the higher value condition.

## Discussion

Here, we asked how OFC neurons represent information about subjective value and whether there might be a causal link between that neural activity and choice behavior. To address this question, we designed a task where the two stimuli representing the choice alternatives were presented sequentially, with the choice occurring later. The most striking findings were that the OFC neurons calculated the difference in value between two offered alternatives, and the selectivity of the neurons changed from emphasizing the difference in values during the second stimulus presentation to emphasizing the chosen value at the time the choice is made. When we inactivated the OFC neurons by injecting the GABA-A agonist muscimol locally, the monkeys’ choice performances were degraded, i.e., the monkeys chose the low value target more frequently when the choice targets were close in value. Thus, OFC neurons carry signals that are related to choice, and the neuronal activity in OFC codes for relative choice value. This neuronal activity is closely related to or even driving the choice behavior, especially when fine distinctions between values must be made for best performance.

Our data showed that many OFC neurons integrate information from different dimensions, here reward size and what we have called workload, which consists of physical effort used to perform the required number of trials and/or the time it takes to perform the trials. The monkey behavior was well fit by the model combining the reward size and the workload. From an earlier behavioral study by Minamimoto et al. it seems that monkeys are more sensitive to delay than to work, at least when work consists of the number of trials^13^. Therefore, perhaps it should not be surprising that Hosokawa et al. reported that OFC neurons are more related to delay than effort (lever weight) in a cost-benefit decision-making task specifically comparing the effects of delay and effort^8^. In our study here, our purpose was to examine whether OFC neurons were sensitive to value, or the individual components that can be considered as different dimensions used to compute relative value, the reward size and workload. Thus, while we cannot know whether one or both of the two covariates, effort and delay, influence the neuronal responses, we have learned that a substantial proportion of OFC neurons represent the value as expressed by single exponential discounting function for reward amount and workload, and these connect value and behavioral performance via a conventional sigmoidal discounting function. Is OFC also involved in the reward value calculation by combining other factors? Blanchard et al. reported that the OFC neurons signal not only reward amount but also informativeness, i.e., whether a choice cue showed the gamble outcome in advance of its delivery^23^. However, they showed that OFC neurons do not integrate these two variables into reward value, suggesting that the OFC does not combine all kinds of factors related to value-based decision-making into the reward value.

Padoa-Schioppa and Assad has reported that the OFC neurons encode the value of chosen goods, that is, chosen value^3^. We also identified many neurons related to the value of the chosen target in the choice phase (53/256, 20.7%). The proportion of the neurons relating to the value difference that formed one of two largest groups in the second target presentation period was the lowest in the choice period (12/256, 4.7%). The results about the chosen target neurons suggest that the activity of OFC neurons encode the chosen value calculated from all of the available alternatives. The task design presenting choice options together makes it difficult to disentangle neuronal activity related to the value comparison between offered alternatives or the value estimation for a stimulus being currently presented. The decision-making schedule task used here presented the alternatives sequentially before choice^23,24^. This task made it straightforward to examine the difference in value of offered alternatives by analyzing the responses to the second target presented. Using this task, we identified a substantial number of neurons related to the value difference between the choice targets.

The number of neurons related to value difference decreased during the fixation period, i.e., when the second target disappeared and decreased even further at the choice phase. Only a small number of value difference neurons identified in the second target presentation period still represented the value difference in the choice phase. These results suggest that the calculation of the difference in value between offered alternatives was already done in the second target presentation period and was no longer needed in the choice phase. The ventro-medial striatum which receives strong projection from OFC and is known to be sensitive to reward value, also, is a likely recipient of this value-difference signal^25–28^.

In this study, we were interested in establishing a causal link between neural activity in the OFC and choice behavior. We showed that the reward values and the difference in value between offered alternatives are encoded in OFC neurons, and that the OFC inactivation affects the choice behavior.

Finally, as mentioned earlier, the OFC projects to a central part of striatum (lateral caudate and ventromedial putamen)^25–28^. Previous studies revealed that the OFC influences action selection by influencing the striatum^29,30^ where some neurons code action as well as reward value^31,32^. Perhaps the value-related signals observed in this study can be used to generate choice within the striatum.

## Methods

### Subjects

Data were obtained from two adult male rhesus monkeys (*Macaca mulatta*; monkey P, ~7.1 kg; monkey H, ~8.4 kg). We trained the monkeys with the reward schedule task, then the decision-making schedule task. The monkeys learned all tasks in 12 months. The experiments were approved by the Animal Care and Use Committee of the University of Tsukuba, and were all conducted in strict accordance with the guidelines for the Care and Use of Laboratory Animals of the University of Tsukuba.

### Experimental conditions

Monkeys sat in a primate chair facing a 22-inch cathode-ray tube (CRT) monitor (CV921X; TOTOKU, Japan) placed 1.0 m from their eyes. Three touch-sensitive bars were attached to the front panel of the primate chair at the level of the monkey’s hand. These bars were referred to as the center bar, and the right and the left choice bars. A liquid reward was dispensed from a stainless tube that was positioned at the monkey’s lips, as previously described^18^. Experiments were conducted in a sound-isolated dark room, and sound was masked further using white noise. Experimental control and data acquisition were performed using the real-time experimental system “REX” adapted for the QNX operating system^33^. Visual stimuli were presented by “Presentation” (Neurobehavioral Systems, Inc., Albany, CA) running on a Windows computer.

### Task procedures

We introduced the decision-making schedule task which was composed of two parts: a decision-making part and a reward schedule part (Fig. 1a and Supplementary Fig. 1). Animals were initially trained to perform simple visual discrimination trials (Supplementary Fig. 1a). The visual discrimination trial started when the animal touched the center bar. Immediately thereafter, a white rectangle visual cue, which we explain later, was presented at the top of the monitor. Then, 800 ms from the onset of the visual cue, a fixation spot (a small white square, 0.17 × 0.17°) was presented at the center of the monitor. The fixation spot was replaced after 400 ms with a red square (WAIT signal, 0.40 × 0.40°). When the red square was present, the monkey had to keep touching the center bar. After 800 ms of WAIT signal presentation, the color of the square changed to green (GO signal). To receive a reward, the monkey had to release the center bar 150–1000 ms after the GO signal. If the monkey released the center bar successfully, the color of the square changed to blue (OK signal), which indicated that the trial had been completed correctly. The visual cue and the square were extinguished after 300 ms from the onset of the OK signal, and a liquid reward was delivered. An error occurred when the monkey released the center bar too early (while the square was red or earlier than 150 ms after the appearance of the GO signal), or did not release the center bar within 1 s of the onset of the GO signal. When the monkey made an error, the visual cue and square were extinguished immediately and the trial was terminated. The inter-trial interval (ITI) was 2 s after a rewarded trial and 3 s after an error.

When the percentage of correct trials for simple visual discriminations exceeded 80%, the reward schedule part was introduced (Supplementary Fig. 1b). In this part, the monkey was required to complete the schedules that were composed of 1, 2, or 4 trials of simple visual discriminations to earn 1, 2, or 4 drops of liquid reward (0.15, 0.30, or 0.60 ml water). During the trials, the visual cue was presented at the top of the monitor. The brightness and length of the visual cue indicated the reward amount and the schedule progress, respectively (Supplementary Fig. 1c). The brightness of the visual cue was proportional to the reward amount: 25% brightness, 1 drop of water; 50% brightness, 2 drops; and 100% brightness (white, 30.19 lux), 4 drops. A previous study reported that the brightness of the visual cue does not affect the neuronal responses in OFC^10^. The length of the visual cue was extended in proportion to the schedule progress. The schedule states were abbreviated as trial number/schedule length: 1/4, 25% of full length (6.06 × 0.60°); 1/2 and 2/4, 50% of full length (12.12 × 0.60°); 3/4, 75% of full length (18.18 × 0.60°); 1/1, 2/2 and 4/4, 100% of full length (24.24 × 0.60°). The trials with the longest cues were reward trials, whereas those with shorter cues were no-reward trials. When the monkey made an error, the same schedule state was repeated.

After learning the reward schedule part, we introduced the decision-making part (Fig. 1a). When the monkey touched the center bar, this part began. At 500 ms from the onset of the fixation spot (a small white square of 0.17 × 0.17°), two kinds of choice target were sequentially presented at the center of the monitor (these targets were called the first and the second target, respectively). Each choice target and fixation point were presented for 500 ms. The brightness and length of the choice target were proportional to the reward amount and schedule length in the reward schedule part, respectively. These two choice targets were picked randomly from the choice target set (Fig. 1b). There were _9_*P*_2_ = 72 pairs of the first and the second targets. After two different choice targets were sequentially presented, these targets reappeared simultaneously, one on each side of a fixation spot in random order (choice phase). To make a decision, the monkey had to touch either the right or the left bar that was on the same side as the chosen target 150–3000 ms after the onset of the choice targets. If the monkey kept touching the chosen bar for 500 ms, the unchosen target and the fixation spot were extinguished. The chosen target was also extinguished after an additional 500 ms, and the chosen reward schedule part began 1 s after a successful choice. If the monkey released the bar before the choice phase or touched the choice bar too early in the choice phase (within 150 ms of the onset of the choice targets), the trial was scored as an early error. If the monkey did not touch either choice bar within 150–3000 ms, the trial was scored as a late error. After these errors, the fixation spot and the choice targets were extinguished and the trial was terminated. A penalty time of 1500 ms occurred after the early and the late error. Then the decision-making part of the trial began again with the same options as the preceding trial.

### Surgery and neurophysiological recording

The location of the OFC was estimated by the 3.0 T magnetic resonance imaging (MRI) (Signa Horizon; GE, Ingenia 3.0 T; Philips). In the surgery, anesthesia was induced with ketamine (4 mg/kg) followed by sodium pentobarbital anesthesia (25 mg/kg). A recording chamber was fixed at an angle of 0° from the median line and the center of this chamber was stereotactically mounted on the left hemisphere based on the MRI (Supplementary Fig. 6) (the center of the recording chamber position, Monkey P: A 33, L 8; Monkey H: A 31, L 7).

We recorded activity from 256 OFC neurons during the decision-making schedule task. All recording conditions were same as the training conditions. Single-unit activity was recorded using tungsten microelectrodes (1.1–1.5 MΩ; Microprobe). In daily experiments, we ran the task while searching for a neuron, and recorded activity from the first neuron we could isolate. We stopped recording when the monkey stopped performing the task for more than 5 min (average trial in each session (mean ± SE); monkey P: 293.7 ± 10.4, monkey H: 309.1 ± 12.6).

### Pharmacological method

To examine the causal role of OFC for the choice behavior, 1–3 μl of muscimol (5 μg/μl, dissolved in normal saline) was locally injected into the recording location of OFC bilaterally (the center of injection site, Monkey P: A 32, L 8 for the left hemisphere, A 33, R 8 for the right hemisphere; Monkey H: A 30, L 7 for the left hemisphere, A 31, R 7 for the right hemisphere). We checked the injection location by MRI.

We took 15 min to inject the muscimol into one hemisphere. After 15 min of muscimol injection, behavioral data were collected. On the next day, the monkeys were injected with saline into the same location as a control. The muscimol injections were conducted four times for both monkeys.

### Analysis of behavioral data

To investigate whether OFC neurons carry signals about both reward value calculated from the reward size and the workload associated with alternatives and the comparison of these values, we trained two adult male rhesus monkeys (monkey P & H) to perform the decision-making schedule task and recorded single neuronal activity from 256 OFC neurons (monkey P: 137, monkey H: 119).

The “R” statistical programming language (R Foundation for Statistical Computing, R Development Core Team, 2004) was used for all statistical analyses.

To estimate the reward value of each choice target, the monkey’s day-by-day choice data were fit by value discounting functions. In standard behavioral models, the widely used functions that account for temporal discounting of future reward are (1) an exponential or (2) a hyperbolic discounting model for reward value^19–21^, which we applied for the workload:

8 $$V = R/e^{kD},$$

9 $$V = R/\left( 1 + kD \right),$$

where *V* is a current reward value (value of the currently presented choice target), *R* is reward amount, *k* is the discounting factor and *D* is the required number of trials to obtain reward. By the following equations, the difference in value between two choice targets was calculated:

10 $$g = V_{1} - V_{2},$$

11 $$g = {\mathrm{log}}\left( {V_{1}/V_{2}} \right),$$

where *V*_1_ is the left target value and *V*_2_ is the right target value. Using this *g*, the monkey’s choice was fit using a standard generalized linear model (GLM) with a binomial link function as follows:

12 $$C = \beta _{0} + \beta_{1} {\mathrm{g}},$$

where *C* is trial-by-trial monkey’s choice (1 indicating choice of left side target and 0 indicating choice of right side target in the choice phase), *β*_0_ is the intercept and *β*_1_ is the coefficient estimated by GLM. We used both value discounting functions to estimate the discounting factor and found that the exponential function was the better model for explanation of monkey’s choice behavior than the hyperbolic function (242/256 [94.5%] choice data during neuronal recordings showed smaller Akaike Information Criterion (AIC) in the exponential model; mean ± SD AIC, exponential model: 83.8 ± 47.8, hyperbolic model: 117.8 ± 58.9; *t* = −7.17, df = 489.33, *p* < 2.73 × 10^−12^, two-tailed *t*-test). We also examined which was the better model, Eqs. 10 or 11, for calculating the value difference. By comparing the value of AIC, 170/256 (66.4%) neurons showed better fit by Eq. 10. Therefore, we estimated the day-by-day discounting factor, *k*, from Eqs. 8, 10, and 12 and used these values for neuronal data analysis.

GLM analysis with a binomial link function was performed for investigating whether the probability of choosing the first and the second target was biased:

13 $$C = \omega _{0} + \omega _{1}R^{\ast} \omega_{2} {\mathrm{FS}},$$

where *C* is trial-by-trial monkey’s choice, *R* is reward value of each target, FS is a term indicating the first/second target (1 indicating the trial focused on the first target and 0 indicating the trial focused on the second target), *ω*_0_ is the intercept and *ω*_1_ and *ω*_2_ are the coefficients.

To examine the relation of the pharmacological inactivation to the choice, we used a generalized linear mixed model (GLMM) with a binomial link function as follows:

14 $$C = \gamma _{0} + \gamma_{1} g^{\ast} \gamma_{2}{\mathrm{Condition}} + \left( 1 | {\mathrm{Subject}} \right),$$

where *C* is trial-by-trial monkey’s choice (1 indicating choice of left side target and 0 indicating choice of right side target in the choice phase), *γ*_0_ is the intercept, *γ*_1_ and *γ*_2_ are the coefficients estimated by GLMM, *g* is the difference in value of two choice targets, Condition is the inactivation condition (1 indicating muscimol treatment and 0 indicating control), and (1|Subject) is the random effect for each monkey.

To examine the session-by-session data in the lowest value difference group (Group 1 in Fig. 4a) of the pharmacological inactivation, following GLMM with a binomial link function was used:

15 $$C = \rho _{0} + \rho_{1} g + \rho _{2} {\mathrm{Condition}} + \left( 1 | {\mathrm{Session}} \right),$$

where *C* is trial-by-trial monkey’s choice (1 indicating choice of left side target and 0 indicating choice of right side target in the choice phase), *ρ*_0_ is the intercept, *ρ*_1_ and *ρ*_2_ are the coefficients estimated by GLMM, *g* is the difference in value of two choice targets, Condition is the inactivation condition (1 indicating muscimol treatment and 0 indicating control), and (1|Session) is the random effect for each session.

As our measure of behavioral performance in the pharmacological experiment, we used reaction time averaged across all the muscimol and the control sessions. Reaction times were defined as the time to touch either the left or the right bar after the choice target appeared simultaneously in the choice phase.

To examine the relationship between the value difference of two choice targets and the reaction time, the following GLMM with a Gaussian link function was used:

16 $${\mathrm{RT}} = \tau _{0} + \tau _{1} g^{\ast} \tau_{2} {\mathrm{Condition}}^{\ast} \tau _{3} {\mathrm{Direction}} + \left( 1 | {\mathrm{Subject}} \right),$$

where RT is the reaction time, *τ*_0_ is the intercept, *τ*_1_, *τ*_2_, and *τ*_3_ are the coefficients estimated by GLMM, *g* is the difference in value of two choice targets, Condition is the inactivation condition (1 indicating muscimol treatment and 0 indicating control), Direction is the choice direction in each trial (1 indicating left bar and 0 indicating right bar), and (1|Subject) is the random effect for each monkey.

### Analysis of neuronal data

The spike counts during the first and the second target presentation period, the fixation period, and the choice phase (500 ms time window) were used for analysis of the recorded neuronal data. All GLM and GLMM analyses were conducted by using all trials we collected in a single session. For each neuron, a correlation between the first target values which were estimated from Eq. 8 and the neuronal responses in the first target presentation period was fit by GLM with a Poisson link function:

17 $${\mathrm{SC}}_{1{\mathrm{st}}} = \theta_{0} + \theta_{1} V_{1},$$

where SC_1st_ is the spike count during the first target presentation period, *θ*_0_ is the intercept, *θ*_1_ is the coefficient, and *V*_1_ is the first target value. We also checked the relation between spike count and reward amount and workload using a following GLM with a Poisson link function:

18 $${\mathrm{SC}}_{1{\mathrm{st}}} = \sigma_{0} + \sigma_{1} R_{1} + {\mathrm{\sigma}}_{2} W_{1},$$

where *σ*_0_ is the intercept, *σ*_1_ and *σ*_2_ are the coefficients, *R*_1_ is the reward amount of the first target, and *W*_1_ is the number of schedules of the first target. We analyzed neurons which showed significant correlation with the reward value of the first target using both Eqs. 17 and 18. To examine which equation is a better model, AIC of these two equations were compared. For over half of neurons (100/173) the spike count was better explained by Eq. 17, which uses the first target value for estimation of the spike counts.

### Reporting summary

Further information on experimental design is available in the Nature Research Reporting Summary linked to this article.

## Supplementary information

Supplementary Information

Reporting Summary

## Data Availability

The data that support the findings of this study are available from the corresponding author on reasonable request.

## Code availability

The original R codes written for the analyses are available from the corresponding author.
