# Supplementary material for: Neurons in the monkey orbitofrontal cortex mediate reward value computation and decision-making
Source: Commun Biol. 2019 Apr 5;2:126. doi: 10.1038/s42003-019-0363-0 (PMC6451015; doi:10.1038/s42003-019-0363-0)
Supplement: Supplementary file 1 — Supplementary Information [file 42003_2019_363_MOESM1_ESM.pdf]

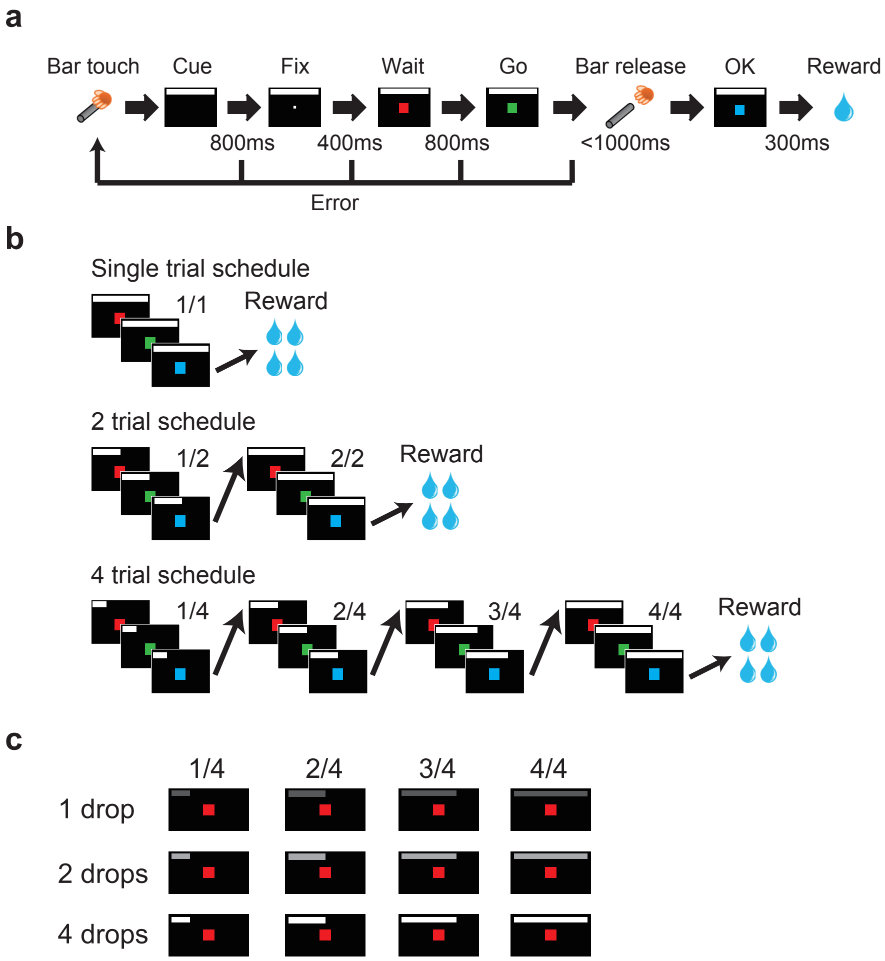

Supplementary Figure 1. Reward schedule part. (a) A visual discrimination trial. When the monkey touched a center bar, the fixation spot appeared. The monkey must release the bar during 150 – 1000 ms after red target changed to green. If the monkey successfully released the bar, the color of the square changed to blue and liquid reward was given. (b) Example of a reward schedule part (with 4 drops of reward). In this part, the monkey was required to perform 1, 2, or 4 repeats of the visual discrimination trial successfully to earn 1, 2, or 4 drops of liquid reward. Throughout the trials, the visual cue was presented at the top of the monitor, and its brightness and length indicated the reward amount and schedule progress, respectively (see Supplementary Fig. 1c). Schedule states were abbreviated as 'trial number / schedule length' (for example, the second trial in a four trial schedule was labeled '2/4'). Trials in which the monkey failed to release the bar within the required time were scored as an error, and the same trial was repeated. (c) Example of cue sets in the reward schedule task (4 trial schedule). The length and brightness of the visual cues were proportional to the schedule progress and reward amount, respectively.

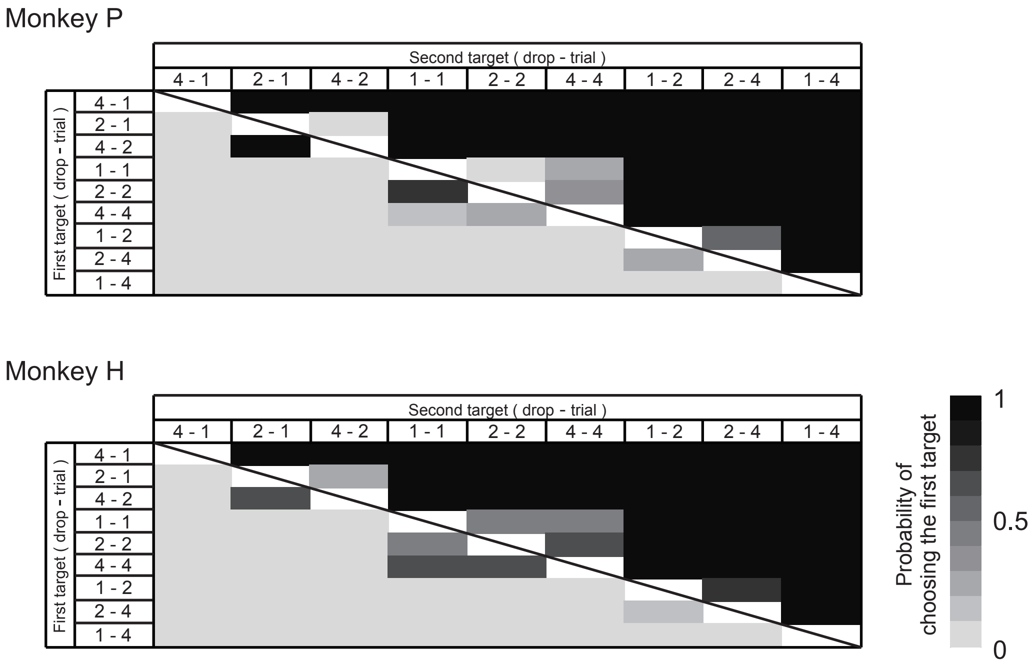

Supplementary Figure 2. Choice probabilities in all combinations of two choice targets. In the labels of the rows (showing the first target) and columns (showing the second target), the reward amount and schedule length of the choice targets are abbreviated as "drop – trial." The labels are ordered according to the ratio of reward amount and schedule length. The gray scale shows the probability of choosing the first target.

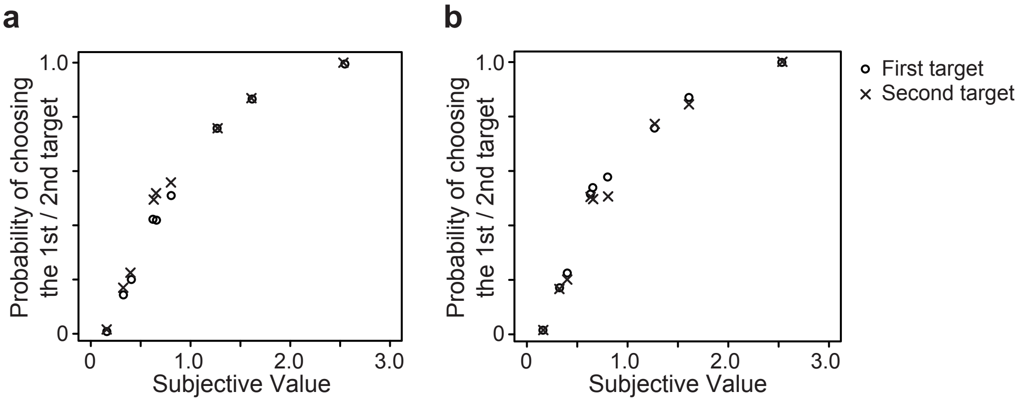

Supplementary Figure 3. Relationship between choice probability and target value. (a and b) The choice probabilities for the first and the second target according to the target values (a) in monkey P and (b) in monkey H.

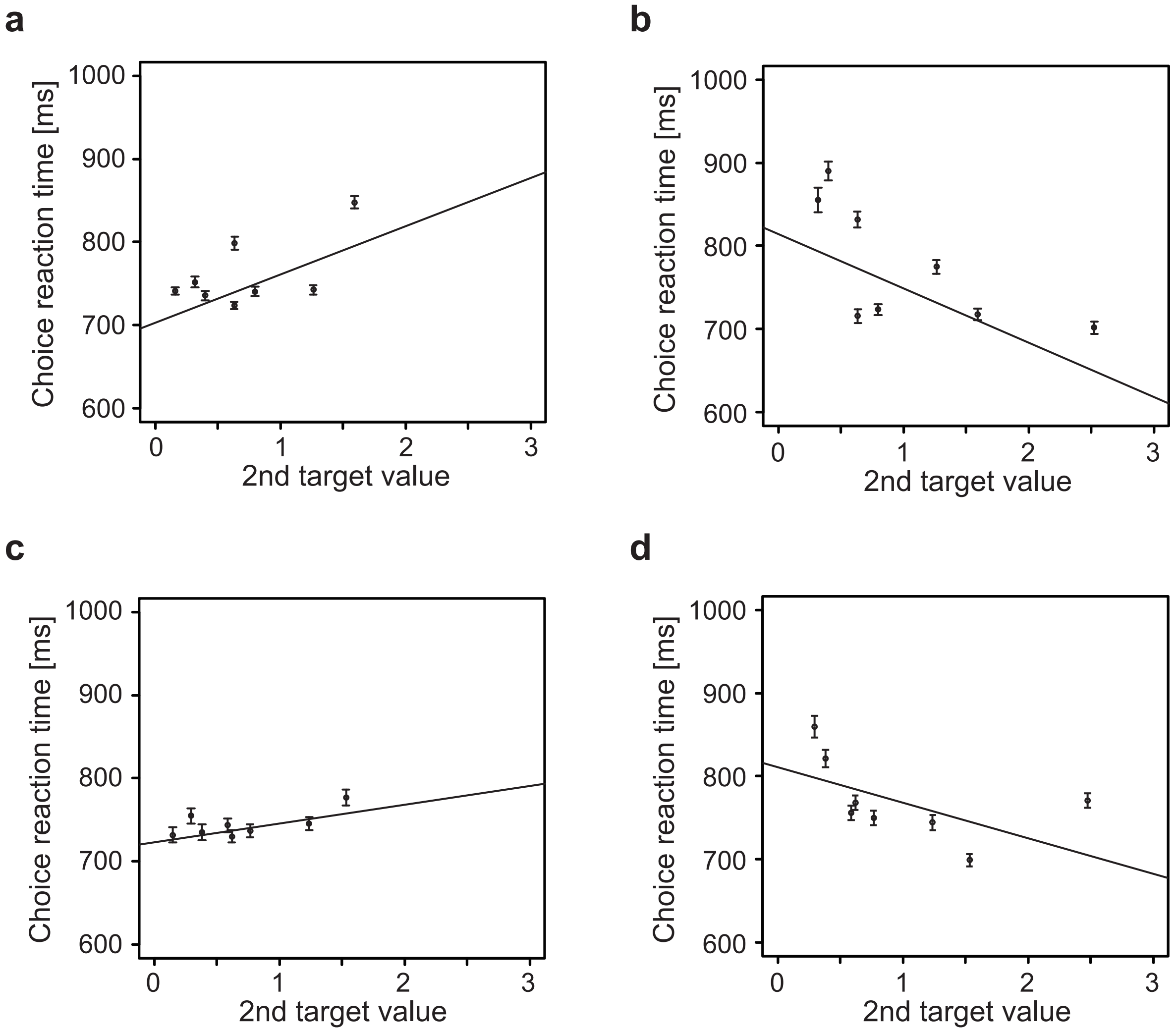

Supplementary Figure 4. Relation between the choice reaction time and the second target value when the largest or smallest value target was presented as the first target. (a and b) Choice reaction time according to the second target value when the target showing (a) 4-1 (drop/ schedule length) (largest value) or (b) 1-4 (smallest value) was presented as the first target in monkey P (linear regression, [4-1] $p < 2.0 \times 10^{-16}$, [1-4] $p < 2.0 \times 10^{-16}$). (c and d) Choice reaction time according to the second target value when the target showing (c) 4-1 or (d) 1-4 was presented as the first target in monkey H. (linear regression, [4-1] $p < 2.0 \times 10^{-16}$, [1-4] $p < 2.0 \times 10^{-16}$).

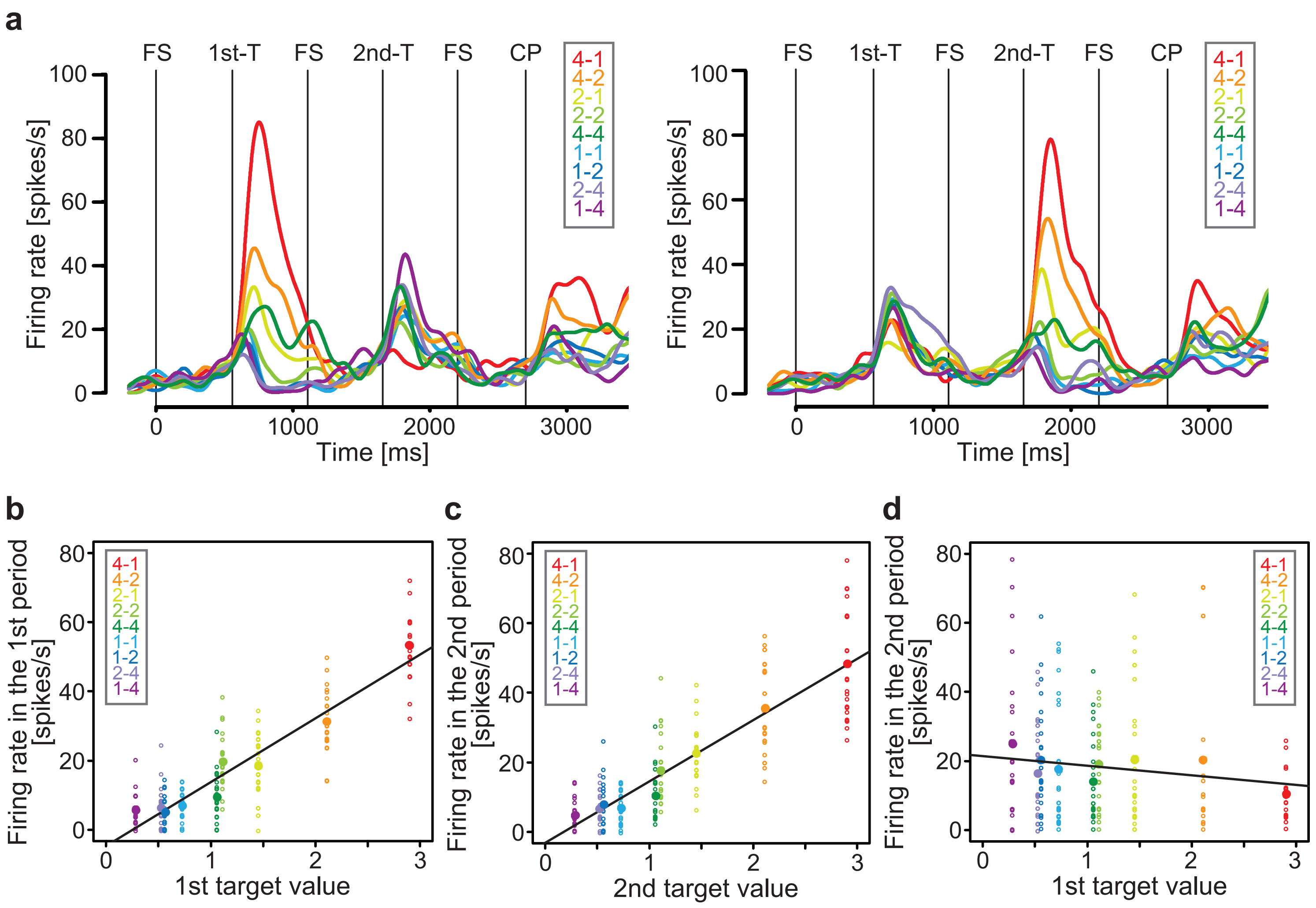

Supplementary Figure 5. An example of neuronal response relating to the value of the currently presented choice target. (a) Left and right panels show the responses classified by the first and the second target, respectively. The same conventions as in Figure 2a. (b) Relationship between the first target value and the firing rate during the first target presentation period (linear regression, n = 215, $R^2 = 0.77$, θ_1: t = 26.50, $p < 2.0 \times 10^{-16}$). Dots: trials, filled large circles: average activity in each target value. (c, d) Relationship between the firing rate during the second target presentation period and (c) the second target value (linear regression, n = 215, $R^2 = 0.71$, α_1: t = 23.01, $p < 2.0 \times 10^{-16}$) or (d) the first target value (linear regression, n = 215, $R^2 = 0.02$, α_1: t = -1.88, $p = 0.06$). The same conventions as in Supplementary Fig. 5b.

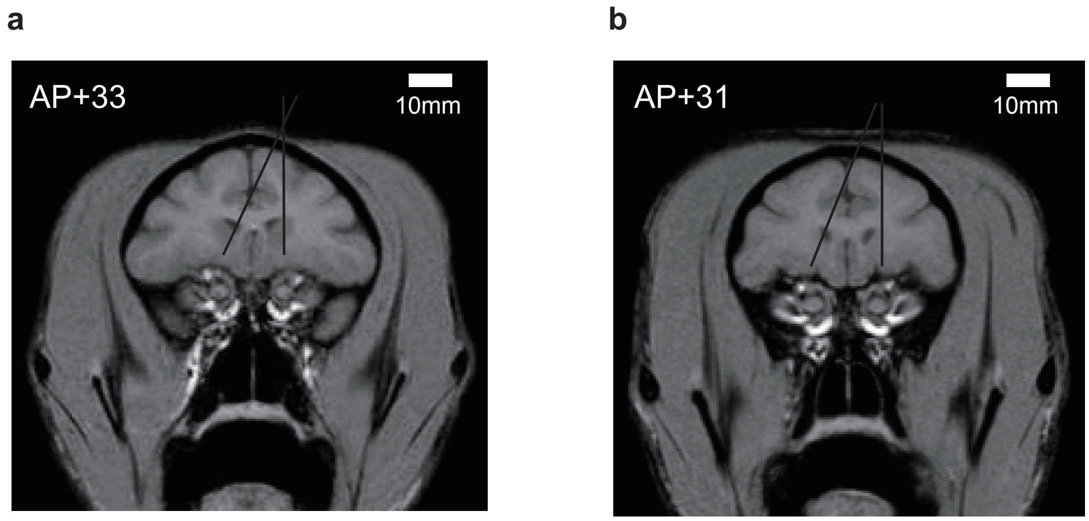

Supplementary Figure 6. Recording and muscimol injection area. (a and b) Frontal T1-weighted MRI section of (a) monkey P and (b) monkey H. The black lines indicate the estimated track of the electrode or the muscimol injection cannula.

Monkey P

	Difference in value					
Group 1	0.104	0.106	0.108	0.120	0.134	0.209
Group 2	0.213	0.226	0.240	0.242	0.346	0.349
Group 3	0.360	0.453	0.469	0.484	0.573	0.589
Group 4	0.693	0.697	0.797	0.831	0.906	0.937
Group 5	0.969	1.039	1.057	1.146	1.177	1.266
Group 6	1.662	1.766	1.874	2.008	2.114	2.234

Monkey H

	Difference in value					
Group 1	0.041	0.046	0.082	0.084	0.122	0.167
Group 2	0.203	0.206	0.244	0.249	0.251	0.289
Group 3	0.370	0.411	0.454	0.495	0.497	0.538
Group 4	0.579	0.620	0.741	0.787	0.823	0.868
Group 5	0.908	0.990	0.992	0.994	1.074	1.076
Group 6	1.573	1.614	1.817	1.862	1.984	2.068

Supplementary Table 1. Difference in value in each group.

Monkey P

	Muscimol		Control		p-value
	High V	Low V	High V	Low V	
Group 1	186	62	336	71	2.0×10^{-2}
Group 2	242	6	387	17	0.11
Group 3	256	0	399	3	-
Group 4	248	2	403	1	-
Group 5	255	0	407	0	-
Group 6	254	0	406	0	-

Monkey H

	Muscimol		Control		p-value
	High V	Low V	High V	Low V	
Group 1	156	108	194	79	1.8×10^{-3}
Group 2	245	21	246	26	0.25
Group 3	262	2	275	0	-
Group 4	269	1	269	2	-
Group 5	266	0	273	0	-
Group 6	266	0	267	5	-

Supplementary Table 2. Number of choosing the high/low value target and p-value of Proportion test between muscimol and control condition.

	Monkey P				Monkey H			
Condition	muscimol		control		muscimol		control	
Chosen target	left	right	left	right	left	right	left	right
Number of trials	738	791	1229	1233	773	844	805	855
Reaction time median (msec)	859	850	730	725	642	723	592	679

Supplementary Table 3. Number of correct trials and median of the reaction time.
