# Supplementary material for: Neurons in the monkey orbitofrontal cortex mediate reward value computation and decision-making
Source: Commun Biol. 2019 Apr 5;2:126. doi: 10.1038/s42003-019-0363-0 (PMC6451015; doi:10.1038/s42003-019-0363-0)
Supplement: Supplementary file 2 — Reporting Summary [file 42003_2019_363_MOESM2_ESM.pdf]

nature research

Corresponding author(s): Munetaka Shidara

Reporting Summary

Nature Research wishes to improve the reproducibility of the work that we publish. This form provides structure for consistency and transparency in reporting. For further information on Nature Research policies, see Authors & Referees and the Editorial Policy Checklist.

Statistical parameters

When statistical analyses are reported, confirm that the following items are present in the relevant location (e.g. figure legend, table legend, main text, or Methods section).

n/a	Confirmed	
☐	☒	The exact sample size (*n*) for each experimental group/condition, given as a discrete number and unit of measurement
☐	☒	An indication of whether measurements were taken from distinct samples or whether the same sample was measured repeatedly
☐	☒	The statistical test(s) used AND whether they are one- or two-sided<br>*Only common tests should be described solely by name; describe more complex techniques in the Methods section.*
☒	☐	A description of all covariates tested
☐	☒	A description of any assumptions or corrections, such as tests of normality and adjustment for multiple comparisons
☐	☒	A full description of the statistics including central tendency (e.g. means) or other basic estimates (e.g. regression coefficient) AND variation (e.g. standard deviation) or associated estimates of uncertainty (e.g. confidence intervals)
☐	☒	For null hypothesis testing, the test statistic (e.g. *F*, *t*, *r*) with confidence intervals, effect sizes, degrees of freedom and *P* value noted<br>*Give P values as exact values whenever suitable.*
☒	☐	For Bayesian analysis, information on the choice of priors and Markov chain Monte Carlo settings
☒	☐	For hierarchical and complex designs, identification of the appropriate level for tests and full reporting of outcomes
☒	☐	Estimates of effect sizes (e.g. Cohen's *d*, Pearson's *r*), indicating how they were calculated
☐	☒	Clearly defined error bars<br>*State explicitly what error bars represent (e.g. SD, SE, CI)*

Our web collection on statistics for biologists may be useful.

Software and code

Policy information about availability of computer code

Data collection	The behavioral task was controlled through custom software "REX" (https://datashare.nei.nih.gov/Laboratory of Sensorimotor Research/LSR Data/lsr/rex/REXdistributions/) and "Presentation" software.
Data analysis	Data analyses were performed using "R" statistical programming language (R Foundation for Statistical Computing, R Development Core Team, 2004).

For manuscripts utilizing custom algorithms or software that are central to the research but not yet described in published literature, software must be made available to editors/reviewers upon request. We strongly encourage code deposition in a community repository (e.g. GitHub). See the Nature Research guidelines for submitting code & software for further information.

Data

Policy information about availability of data

All manuscripts must include a data availability statement. This statement should provide the following information, where applicable:

- Accession codes, unique identifiers, or web links for publicly available datasets
- A list of figures that have associated raw data
- A description of any restrictions on data availability

The data that support the findings of this study are available from the corresponding author on reasonable request.

Field-specific reporting

Please select the best fit for your research. If you are not sure, read the appropriate sections before making your selection.

☒ Life sciences ☐ Behavioural & social sciences ☐ Ecological, evolutionary & environmental sciences

For a reference copy of the document with all sections, see nature.com/authors/policies/ReportingSummary-flat.pdf

Life sciences study design

All studies must disclose on these points even when the disclosure is negative.

Sample size	We used 2 animals, which is minimum and standard number for non-human primate studies. The number of recorded cells and the number of trials ran for each cell (sample size) were not pre-determined using any statistical method.
Data exclusions	No data were excluded from the analyses.
Replication	We recorded OFC neurons or behavioral data from two animals, and all data were consistent between them.
Randomization	Muscimol and control sessions in the pharmacological experiment were interleaved in the experiments, but the order was not determined using a formal randomization.
Blinding	We did not do blinding during data collection and analysis because we recorded OFC neuronal activities from all possible location of the recording chamber and used all the neuronal data we could isolate for the analyses, and in the pharmacological experiment, both muscimol and control sessions were tested to the same monkey.

Reporting for specific materials, systems and methods

Materials & experimental systems

n/a	Involved in the study
☒	☐ Unique biological materials
☒	☐ Antibodies
☒	☐ Eukaryotic cell lines
☒	☐ Palaeontology
☐	☒ Animals and other organisms
☒	☐ Human research participants

Methods

n/a	Involved in the study
☒	☐ ChIP-seq
☒	☐ Flow cytometry
☒	☐ MRI-based neuroimaging

Animals and other organisms

Policy information about studies involving animals; ARRIVE guidelines recommended for reporting animal research

Laboratory animals	Two adult male rhesus monkeys
Wild animals	Wild animals were not used in this study.
Field-collected samples	Field-collected samples were not used in this study.
